# Prevalence and correlates of workplace violence against medical assistants in Germany: a cross-sectional study

**DOI:** 10.1186/s12913-023-09331-9

**Published:** 2023-04-10

**Authors:** Viola Mambrey, Stefanie Ritz-Timme, Adrian Loerbroks

**Affiliations:** 1 Institute of Occupational, Social and Environmental Medicine, Centre for Health and Society, Faculty of Medicine, University of Duesseldorf, Moorenstraße 5, Düsseldorf, 40225 Germany; 2 Institute of Legal Medicine, University Hospital Duesseldorf, Moorenstraße 5, Düsseldorf, 40225 Germany

**Keywords:** Ambulatory care, Epidemiology, Health Personnel, Medical Assistants, Prevalence, Workplace violence

## Abstract

**Background:**

Workplace violence is widespread, but studies on workplace violence against health professions in outpatient settings are sparse. We aimed to examine, for the first time, the prevalence of workplace violence against medical assistants as well as potential sociodemographic, occupational and health-related correlates of the exposure to workplace violence.

**Methods:**

We used data from a survey (03–05/2021) among medical assistants in Germany (*n* = 424). We assessed the 12–month prevalence (yes/no) of verbal violence, physical violence, and sexual harassment as well as the types of perpetrators of workplace violence. Further, information was gathered on sociodemographic (e.g., age, educational level), occupational (e.g., years in job), and mental health-related factors (i.e., anxiety, depression). The 12–month prevalences of the different types of workplace violence were merged into a single variable (“any workplace violence” vs. none) for association analysis. We ran multivariable Poisson regression models to examine potential associations between sociodemographic and occupational correlates (i.e., independent variables) with any workplace violence as dependent variable and in addition between any workplace violence (independent variable) and dichotomized mental health as dependent variable.

**Results:**

Overall, 59.4% of the medical assistants reported verbal violence, 5.9% reported physical violence, 3.8% reported sexual harassment, and 60.1% reported any workplace violence in the previous 12 months. Patients were reported to be the main perpetrators, followed by patients’ relatives. Younger age, being single, and working in a medical care center were sociodemographic and occupational correlates of workplace violence (PRs ≥ 1.27). Workplace violence was significantly associated with mental health variables (PRs ≥ 1.72).

**Conclusion:**

Medical assistants experience workplace violence, in particular verbal violence. To devise preventive measures, prospective studies are needed to confirm the potential risk groups for workplace violence and the potential mental health sequels of workplace violence observed in our study.

**Supplementary Information:**

The online version contains supplementary material available at 10.1186/s12913-023-09331-9.

## Introduction

Aggression and violence against healthcare workers represent a global challenge [1]. Workplace violence (WPV) can be defined as "incidents where staff are abused, threatened, or assaulted in circumstances related to their work, […], involving an explicit or implicit challenge to their safety, well-being, or health" [2]. WPV can take various forms, which often co-occur, and may be conceptualized in terms of physical violence (e.g., hitting, kicking, pushing, or biting), verbal violence (e.g., threatening, insulting, or yelling) or sexual harassment (e.g., suggestive remarks, intrusive staring, or unwanted touching) [2, 3]. A meta-analysis found the 12-month prevalence of any type of WPV reported by health care workers in inpatient and outpatient settings worldwide to equal 61.9% [1]. With regard to specific types of WPV, a systematic review on WPV against health care workers in outpatient settings showed prevalence estimates of WPV perpetrated by patient and patient families to range between 42.1% and 94.3% for verbal violence, between 0.5% and 15.9% for physical violence, and from 0.2% to 9.3% for sexual harassment [4]. The estimated prevalence’s of WPV among health care workers vary considerably between studies and are thought to depend, amongst others, on the definition of WPV applied, the country, the work settings (e.g., outpatient care, inpatient care) and the type of health care profession (e.g., nurse, physician) [1, 5, 6]. With regard to perpetrators within the health care system a distinction is often made between patients, patients’ relatives, colleagues, and supervisors. Previous studies identified patients and their relatives as the main perpetrators of WPV [5].

A range of sociodemographic and occupational factors has been found to be associated with the experience of WPV including younger age, a higher number of working hours, and fewer years of work experience [1, 7]. WPV has also been found to be associated with poor health outcomes among health care workers, such as anxiety and depressive symptoms [6].

The majority of existing studies has focused on health care workers in inpatient settings, and only a few studies address WPV in outpatient settings [8–14]. In Germany, outpatient care is a central pillar of the health care system, as outpatient treatment is utilized by 93% of the population at least once a year [15]. The largest occupational group in outpatient care in Germany are medical assistants (MAs) and the majority of MAs works in outpatient care [16]. MAs support physicians in their daily work by taking on many patient-related clinical tasks (e.g., blood sampling, administering injections, wound care and performing X-rays or electrocardiography) as well as administrative tasks (e.g., managing practice procedures, accounting, documenting patient histories) [17]. MAs are also in charge of the reception desk and answering the telephone, which entails a high level of interaction with patients and patients’ relatives, which has been identified as a probable risk factor of WPV [14, 18]. Outpatient physician practices in Germany are characterized by work in small teams. The usually small team size in outpatient care facilities may carry specific risks relevant to WPV [4, 19]. For instance, options for protection of staff (e.g., through security guards) against WPV generated by patients or patients’ relatives may be limited. Further, in small companies a formal contact person to turn to in case of WPV is lacking (e.g., employee representatives) and it may be challenging to turn to colleagues and/or the supervisor due to social dependencies: the daily work of MAs is characterized by strong collaboration within the team [20] and complaints in small teams against colleagues or the supervisor (especially when those are the perpetrators) seem difficult as it is conceivable that they may induce or aggravate social tensions, hamper future collaboration and may even jeopardize one’s job. With regard to the latter, it needs mentioning that the supervisor in outpatient practices is often also the employer and that the protection against dismissal is low in small companies (< 10 employee) in Germany [Dismissal Protection Act (§ 23 KSchG)]. Even though inpatient and outpatient care settings share the interaction with patients and patients’ relatives, the above-mentioned specificities of work tasks, social work environments and legal peculiarities may affect the prevalence of experienced WPV as well as the sociodemographic and occupational correlates of WPV [4].

So far, MAs have been excluded from epidemiological research on WPV. However, they might represent a high-risk group as MAs are the first point of contact for patients, work in close patient contact and report to experience low recognition by supervisors and society [14, 18, 20, 21].

Prior work on WPV in outpatient health care staff is characterized by several knowledge gaps or inconsistent findings which we seek to address. First, earlier research in outpatient care seems to have taken a piecemeal approach by having considered solely patients and their relatives as perpetrators [4]. As a result, estimates related to colleagues and supervisors as a potential source of WPV are unknown. Second, while many sociodemographic and occupational factors have been examined as potential correlates in prior work, the evidence remains inconsistent (e.g. regarding migration background, number of coworkers) [1, 8–10, 12, 22]. Identifying profession-specific correlates and thus potential risk factors is necessary though in order to devise appropriate prevention measures for MAs. Finally, mental health sequels have been examined in studies amongst hospital and inpatient care staff [6], but evidence is sparse for the outpatient care setting [8, 9, 12].

We sought to address the above-mentioned knowledge gaps and to contribute data to areas where findings were mixed by 1) providing information on the 12-month prevalence of WPV among MAs, including the types of perpetrators; 2) exploring potential sociodemographic and occupational correlates of WPV and 3) examining associations between WPV and mental health.

## Methods

### The study population

We utilized follow-up data from a cohort study among MAs in Germany investigating psychosocial working conditions and health. Baseline questionnaire data were collected between September 2016 and April 2017 [23, 24]. MAs currently in training or holding a MA degree were included in the study at baseline. There were no further inclusion or exclusion criteria. Various associations and organizations helped to recruit participants throughout Germany. Multiple communication channels were used including the distribution of flyers by means of the members magazine of the Association of Medical Professions (Verband Medizinischer Fachberufe [VMF] e.V.), which is the professional association of MAs in Germany. The study was further advertised by the Association of Statuary Health Insurance Physicians via home pages, internal distribution or direct forwarding to medical practices or professional MA schools (for further information see [23]). In total, 944 MAs completed the baseline questionnaire.

The follow-up assessment, which included questions regarding WPV for the first time, was conducted between March 2021 and May 2021 (*n* = 537 MAs, response rate = 56.9%) [25]. The current analysis was restricted to follow-up data of those who reported to be in current employment as a MA (*n* = 424) as opposed to, for instance, reported employment in another profession, parental leave, unemployment or retirement. Our study was approved by the Ethics Committee of the Medical Faculty of the Heinrich-Heine-University of Düsseldorf (ethic registration number:2019-819_2).

### Survey instruments

#### Workplace violence

WPV was measured by an instrument which we devised based on a questionnaire provided by the International Labor Organization (ILO) measuring specifically experiences of violence among health care staff [26] [for the administered questionnaire please see Additional file 1]. We discussed the items of the questionnaire in the study team and decided to shorten the detailed questionnaire. We included the definitions of the different types of WPV and the following items from the original ILO questionnaire: 12–month prevalence by type [no/yes], perpetrators [patient, relatives of patient, staff member, supervisor, external colleague, general public, others] of physical violence, verbal violence and sexual harassment, respectively, the frequency [all the time, sometimes, once] of verbal violence and sexual harassment in the previous 12 months and an item concerning the use of a weapon during a physical violence attack [physical violence without a weapon/physical violence with a weapon]. The frequency of physical violence was not assessed in the original ILO questionnaire. However, we added this item to gather the respective information and to standardize the structure of the questionnaire. In addition, we decided to exclude external colleagues and general public as perpetrators from the response option to adapt the response option to the outpatient care setting in Germany, which is characterized by small professional teams with no direct external colleagues and limited contact with the general public beyond contact with patients and their relatives.

The ILO questionnaire is not available in German, therefore the respective items were translated to German by the first author and the translation was checked by a fellow researcher (AD). The German-language questionnaire was then refined through discussion with experts from the Association of Medical Professions (VMF e.V.) in terms of relevance and completeness. The experts of the association are MAs themselves and are in close contact with MAs in order to adequately represent their interests and have experience in conducting surveys among MAs. The experts suggested to adapt the definitions provided in the original ILO questionnaire on the different types of WPV (i.e., physical and verbal violence, sexual harassment) slightly to ensure a more informal tone in order to achieve a better understanding among participants. The definitions provided examples of WPV (e.g., hitting, pushing) which were based on findings on violence in informal care [27]. Moreover, it was suggested to adapt the response options regarding the frequency of WPV in the previous 12 months from non-specific (i.e., all the time, sometimes, once) to more specific time periods (e.g., almost daily, monthly, once in a quarter) and to add an item assessing the type of sexual harassment experienced by MAs. Therefore, the type of sexual harassment (e.g., sexually suggestive remarks and jokes; intrusive or intimidating stares or suggestive glances) was assessed based on the legal definition in paragraph 3 Sect. 4 of the German General Equal Treatment Act (§ 3 Abs. 4 AGG), provided by the Federal Anti-Discrimination Agency [28].

The instrument was then refined by means of cognitive interviews with MAs recruited through the VMF e.V. (*n* = 4), which explored the overall impression, understanding, and completeness. The final instrument comprises 11 items [see Additional file 1].

#### Correlates of workplace violence

We assessed 16 self-reported possible sociodemographic, occupational and health-related correlates of WPV. The selection of potential correlates was based on the literature on factors associated with WPV in healthcare professions [1, 7].

Sociodemographic variables included:

- Age in years, categorized into tertiles (≤ 42, 43–52, ≥ 53)
- Sex categorized into female and non-female (i.e. male or non-binary)
- Partnership status was defined as being single versus in a partnership
- The highest educational level was operationalized according to the German school system as low (secondary modern school qualification [‘Haupt-/Volksschulabschluss’]), intermediate (secondary school level I certificate ([‘Mittlere Reife’]) or high (general qualification for university entrance [‘Abitur’] or entrance qualification limited to universities of applied sciences [‘Fachhochschulreife’])
- Gross income grouped into four categories (≤ 1999€, 2000–2499€, 2500–2999€, ≥ 3000€)
- Migration background—categorized into “yes” and “no”- was considered to be present if one of the following conditions was met: I) both parents born abroad or II) participant born abroad and at least one parent born abroad or III) mother tongue is not German [29]

Occupational variables included:

- Total number of years worked as a MA since completion of MA vocational training (“years in job”), categorized into three roughly equalized sized groups (≤ 18, 19–29, ≥ 30)
- Number of mean working hours per week including overtime, categorized into three roughly equalized sized groups (≤ 30, 31–39, ≥ 40)
- Type of employer (general practitioner [GP] practice, specialist practice, medical care center, hospital/clinic, others)
- Employment status according to one’s contract (“full-time” generally refers to around 35–42 h per week vs “part time/mini-job” lower number of hours than the full-time contract)
- Leadership position (“yes”/“no”)
- Number of MAs employed in the respective practice (categorized into three roughly equalized sized groups: 1–3 MAs, 4–6 MAs, ≥ 7 MAs)
- Number of physicians in the respective practice (categorization into tertiles was not feasible based on the distribution. Therefore, this variable was categorized into four roughly equalized sized groups: 1 physician, 2 physicians, 3–4 physicians, ≥ 5 physicians)
- Practice location based on the following definitions: rural area (< 20,000 inhabitants), small city (20,000 to 100,000 inhabitants), or large city (> 100,000 inhabitants)

Health variables included:

- Anxiety was assessed using the established short version of the Generalized Anxiety Disorder questionnaire (GAD-2) [30] which has been shown to be a reliable screening tool for generalized anxiety disorder [31]. The GAD-2 inquires after the frequency of symptoms with a 4-point Likert scale varying between “not at all” (0) and “almost every day” (3). The score can range from 0 to 6 and the cut-off for anxiety was set at ≥ 3 [30]. The internal consistency in our sample was 0.86 (Spearman-Brown coefficient).
- Depressive symptoms were assessed by the short version of the Patient Health Questionnaire (PHQ-2) [30], which has been validated in previous studies and shows good psychometric properties and convergent validity with other established measures such as the Hospital Anxiety and Depression Scale [32]. Responses are given on a 4-point Likert scale assessing the frequency of symptoms from “not at all” (0) to “almost every day” (3). The score can range from 0 to 6 and the cut-off for depression is 3 and above [30]. The internal consistency in our sample for PHQ-2 scale was 0.79 (Spearman-Brown coefficient).

### Statistical analysis

Descriptive analyses built on the estimation of the prevalences and distributions of the variables of interest. The number of cases reporting physical violence (*n* = 25, 5.9%) and sexual harassment (*n* = 16, 3.8%) were low and thus the statistical power seemed very limited when these dependent variables are considered separately in analyses exploring correlates. We therefore merged the variables measuring physical and verbal violence, and sexual harassment into a composite variable called “any WPV” (any affirmative response vs. none) for the association analysis. Association analysis included the following combinations of independent and dependent variables:

1. Independent variable: Sociodemographic variables (dichotomous or categorical)
  Dependent variable: Any workplace violence (dichotomous)
2. Independent variable: Occupational variables (dichotomous or categorical)
  Dependent variable: Any workplace violence (dichotomous)
3. Independent variable: Any workplace violence (dichotomous)
  Dependent variable: Mental health variables [i.e., anxiety and depression] (dichotomous)

The odds ratios derived from binomial logistic regressions may overestimate true estimates in epidemiological studies and log-binomial regression has been recommended instead [33]. We therefore initially ran log-binomial regression models. However, one of those models failed to converge. Therefore, as recommended [33], we examined potential associations using Poisson regression with robust estimators and a log-link function which yields prevalence ratios (PR) and corresponding 95% confidence intervals (CI) [33]. The significance level was set to an alpha level of 0.05. For each independent variable a separate Poisson regression model was computed. We ran unadjusted models and further initially intended to adjust these models additionally for age, average working hours per week, sex and years in job. These variables have been identified as potential confounders in previous studies [1, 7]. However, due to the very low number of non-female participants (*n* = 6, 1.4%), we did not adjust for sex and removed sex as a determinant from the association analysis. Years in job was not considered as a cofounder due to strong correlations with age (Pearson correlation coefficient = 0.86). Therefore, models were adjusted for age and average working hours per week.

We also explored a potential association between the frequency of WPV (almost daily, weekly, monthly, once in a quarter, once in a year) and mental health variables. Due to the low number of cases who experienced physical violence (*n* = 25, 5.9%) and sexual harassment (*n* = 16, 3.8%) we based our analysis only on the frequency of verbal violence. A Poisson regression model was computed with “frequency of verbal violence” as independent variable and dichotomized health related variables as dependent variables adjusted for age and mean working hours per week. The Mantel–Haenszel test was applied to evaluate trends.

The data were analyzed using IBM SPSS Statistics 25. Missing values were observed only for the variables practice location and current employer with each one missing (0.2%), for employment status with eight missings (1.9%), for number of MAs in the employing practice with 29 missing (6.8%), and 31 missing (7.3%) for number of physicians in the employing practice.

## Results

### Sample population

Characteristics of the study population are shown in Table 1. The mean age of the participants was 46.8 years (standard deviation (SD) = 10.4 years) and 98.6% of the MAs reported to be female. Being in a partnership was reported by 88.2% of the participants and 4.7% reported to have a migration background. The mean of years working in the job as an MA was 23.9 years (SD = 11.3 years) with an average weekly working time of 33.4 h (SD = 8.7 h). Participants mainly worked in outpatient care (85.8%) (i.e., GP practice, specialist practice or medical care center). Most participants worked in large cities (42.8%) followed by small cities (36.9%) and rural areas (20.3%). Anxiety and depressive symptoms were reported by 19.8% and 20.8% of the MAs, respectively.

**Table 1:** Description of the sample (*n* = 424)

**Characteristic**		
	***n***	**(%)**
Age, mean (M), standard deviation (SD), (min–max)	46.8	(10.4), (25–70)
≤ 42	144	(34.0)
43—52	130	(30.7)
≥ 53	150	(35.4)
Sex		
Female (vs. non-female^a^)	418	(98.6)
Partnership		
Yes (vs. no)	374	(88.2)
Highest school degree^b^		
Low	25	(5.9)
Intermediate	315	(74.3)
High	84	(19.8)
Gross salary (€)		
≤ 1999	121	(29.7)
2000–2499	101	(24.8)
2500–2999	89	(21.8)
≥ 3000	97	(23.8)
Did not want to disclose	16	(3.8)
Migration background		
Yes (vs. no)	20	(4.7)
Years in job, M (SD), (min–max)	23.9	(11.3), (3–53)
≤ 18	145	(34.2)
19 – 29	135	(31.8)
≥ 30	144	(34.0)
Average weekly working hours, M (SD), (min–max)	33.4	(8.7), (3–60)
≤ 30	158	(37.3)
31 – 39	135	(31.8)
≥ 40	131	(30.9)
Current employer ^c^		
General practitioner practice	162	(38.3)
Specialist practice	158	(37.4)
Medical care center	44	(10.4)
Hospital/clinic	33	(7.8)
Others	26	(6.1)
Employment status ^d^		
Full-time	208	(50.0)
Part time/Mini-job	208	(50.0)
Leadership position		
Yes	206	(48.6)
Number of medical assistants in the practice, M (SD) ^e^	8.3	(15.6)
1–3	114	(28.9)
4–6	147	(37.2)
≥ 7	134	(33.9)
Number of physicians in the practice, M (SD) ^f^	3.7	(3.8)
1	104	(26.5)
2	100	(25.4)
3–4	95	(24.2)
≥ 5	94	(23.9)
Practice location ^c^		
Large city (> 100,000 inhabitants)	181	(42.8)
Small city (> 20,000 inhabitants)	156	(36.9)
Rural area (< 20,000 inhabitants)	86	(20.3)
Anxiety (According to GAD-2)^g^		
Yes	84	(19.8)
Depression (According to PHQ-2)^h^		
Yes	88	(20.8)

^a^non-female includes male and non-binary

^b^low: secondary school qualification (‘Haupt-/Volksschulabschluss’); intermediate: secondary school level I certificate (‘Mittlere Reife’); high: general qualification for university entrance (‘Abitur’) or entrance qualification limited to universities of applied sciences (‘Fachhochschulreife’)

^c^one missing

^d^eight missings (1.9%)

^e^29 missings (6.8%)

^f^31 missings (7.3%)

^g^generalized anxiety disorder questionnaire (GAD-2)

^h^patient health questionnaire (PHQ-2)

### Descriptive analysis

As shown in Table 2, 60.1% of the participants reported to have experienced at least one type of WPV in the preceding 12 months (any WPV). Verbal violence was reported to be experienced by 59.4% of the MAs during the preceding 12 months. Of those affected, 80.5% reported to have experienced verbal violence weekly to once in a quarter. The most frequently reported perpetrators of verbal violence were patients (82.5%), followed by patient relatives (46.8%), colleagues (19.4%), supervisors (19.0%) and others (2.4%). In our sample, 5.9% of the MAs reported to have experienced physical violence during the preceding 12 months. Physical violence was reported to have been experienced predominantly once in a quarter to once in a year (92%). Although the number of absolute cases is low, it can be observed that mainly patients were reported as perpetrators. One participant reported that a weapon was used during a physical violence incident. During the preceding 12 months sexual harassment was reported by 3.8% of the participants, with most of them (87.6%) reported to have experienced sexual harassment monthly to once in a quarter. The data indicate that mainly patients were reported as perpetrator. Participants who reported sexual harassment (*n* = 16) specified to experience sexually suggestive remarks and jokes (*n* = 13), intrusive or intimidating stares or suggestive glances (*n* = 10), unwanted touches (e.g., patting, caressing, hugging; *n* = 8) and solicitation of unwanted intimate or sexual acts (e.g., "Sit on my lap"; *n* = 3).

**Table 2:** Twelve-month prevalence of workplace violence (WPV) among medical assistants, frequency of WPV and perpetrators of WPV by type of WPV (verbal, physical and sexual) in absolute numbers and percentages (*n* = 424)

	**Verbal violence**		**Physical violence**		**Sexual harassment**		**Any WPV**^**a**^	
	**n**	**(%)**	**n**	**(%)**	**n**	**(%)**	**n**	**(%)**
**Twelve-month prevalence**	252	(59.4)	25	(5.9)	16	(3.8)	255	(60.1)
**Frequency of WPV**								
Once in a year	29	(11.5)	15	(60.0)	1	(6.3)		
Once in a quarter	87	(34.5)	8	(32.0)	7	(43.8)		
Monthly	63	(25.0)	0		7	(43.8)		
Weekly	53	(21.0)	1	(4.0)	1	(6.3)		
(Almost) daily	20	(7.9)	1	(4.0)	0			
**Perpetrator**^b^								
Patients	208	(82.5)	21	(84.0)	15	(93.8)		
Relatives of patients	118	(46.8)	5	(20.0)	1	(6.3)		
Colleagues	49	(19.4)	1	(4.0)	1	(6.3)		
Supervisors	48	(19.0)	1	(4.0)	0			
Others	6	(2.4)	2	(8.0)	0			

^a^any WPV: 12-month prevalence of verbal violence, physical violence or sexual harassment merged into a single variable (any affirmative response vs. none)

^b^multiple answers possible

### Association analyses

Overall, only a few sociodemographic and occupational correlates were associated with any WPV (see Table 3 and 4, respectively). Younger participants (≤ 42 years) reported any WPV 1.3 times more often than older participants (≥ 53 years) (95%CI 1.06–1.52). The same held true for participants who reported not to be in a partnership. MAs working in medical care centers reported significantly more often to have experienced any WPV in comparison to MAs working in general practitioner practices (PR = 1.36 95%CI = 1.10–1.68, see Table 4).

**Table 3:** Association of sociodemographic variables with any workplace violence (WPV) among medical assistants

Characteristic	Any WPV^a^			
	**Model I**^b^		**Model II**^**c**^	
	**PR**	**95% CI**	**PR**	**95% CI**
Age				
≥ 53	1.0	ref	1.0	ref
43—52	0.98	0.79-1.22	0.97	0.79-1.21
≤ 42	**1.28**	1.07-1.53	**1.27**	1.06-1.52
Partnership				
Yes	1.0	ref	1.0	ref
No	**1.34**	1.12-1.60	**1.31**	1.10-1.57
Highest school degree^d^				
Low	1.0	ref	1.0	ref
Intermediate	1.01	0.71-1.44	1.01	0.72-1.41
High	1.08	0.74-1.58	1.04	0.72-1.49
Gross salary (€)				
≤ 1999	1.0	ref	1.0	ref
2000–2499	1.06	0.86-1.31	0.98	0.79-1.21
2500–2999	1.04	0.83-1.29	0.89	0.70-1.13
≥ 3000	0.93	0.74-1.17	0.81	0.62-1.06
Migration background				
No	1.0	ref	1.0	ref
Yes	1.21	0.91-1.60	1.13	0.87-1.49

Prevalence ratio (PR) and 95% confidence interval (CI); ref = reference category

^a^any WPV: 12-month prevalence of verbal violence, physical violence or sexual harassment merged into a single variable (any affirmative response vs. none)

^b^unadjusted

^c^additionally adjusted for age and average working hours per week

^d^low: secondary modern school qualification (‘Haupt-/Volksschulabschluss’); intermediate: secondary school level I certificate (‘Mittlere Reife’); high: general qualification for university entrance (‘Abitur’) or entrance qualification limited to universities of applied sciences (‘Fachhochschulreife’)

number in bold = significant *p*-value < 0.05

**Table 4:** Association of occupational variables with any workplace violence (WPV) among medical assistants

Characteristic	Any WPV^a^			
	**Model I**^b^		**Model II**^**c**^	
	**PR**	**95% CI**	**PR**	**95% CI**
Years in job				
≥ 30	1.0	ref	1.0	ref
19 – 29	0.95	0.77-1.17	0.96^e^	0.78-1.12
≤ 18	1.13	0.94-1.36	1.13^e^	0.95-1.36
Average weekly working hours				
≥ 40	1.0	ref	1.0	ref
31 – 39	1.11	0.92-1.33	1.11	0.92-1.33
≤ 30	0.87	0.71-1.07	0.87	0.71-1.07
Current employer				
General practitioner practice	1.0	ref	1.0	ref
Specialist practice	1.04	0.86-1.26	1.06	0.88-1.28
Medical care center	**1.38**	1.12-1.71	**1.36**	1.10-1.68
Hospital/clinic	1.25	0.96-1.62	1.22	0.94-1.58
Others	0.86	0.56-1.32	0.85	0.54-1.32
Employment status				
Full-time	1.0	ref	1.0	ref
Part time/mini-job	1.05	0.90-1.24	1.23	0.95-1.59
Leadership position				
No	1.0	ref	1.0	ref
Yes	1.12	0.96-1.31	1.10	0.94-1.29
Number of medical assistants in the employing practice				
≥ 7	1.0	ref	1.0	ref
4–6 (vs. ≥ 7)	1.01	0.82-1.24	0.98	0.80-1.20
1–3 (vs. ≥ 7)	0.96	0.78-1.17	0.96	0.78-1.17
Number of practitioners in the employing practice				
≥ 5	1.0	ref	1.0	ref
3–4 (vs. ≥ 5)	1.13	0.89-1.43	1.09	0.86-1.38
2 (vs. ≥ 5)	1.22	0.97-1.53	1.16	0.93-1.45
1 (vs. ≥ 5)	0.96	0.74-1.24	0.95	0.74-1.22
Practice location^d^				
Large city	1.0	ref	1.0	ref
Small city	0.86	0.72-1.03	0.87	0.73-1.04
Rural area	0.93	0.75-1.14	0.95	0.77-1.16

Prevalence ratio (PR) and 95% confidence interval (CI); number in bold = significant *p*-value < 0.05

^a^any WPV: 12-month prevalence of verbal violence, physical violence or sexual harassment merged into a single variable (any affirmative response vs. none)

^b^unadjusted

^c^additionally adjusted for age and average working hours per week

^d^large city: > 100,000 inhabitants; small city: > 20,000 inhabitants; rural area: < 20,000 inhabitants

^e^due to strong correlation between years in job and age, age was excluded from adjustment

Associations of any WPV with mental health variables among MAs are shown in Table 5. We observed significant positive associations of any WPV (vs none) with both anxiety (PR = 1.85 95%CI = 1.16–2.95) and depression (PR = 1.72 95%CI = 1.11–2.67).

**Table 5:** Association of any workplace violence (WPV) with health-related variables among medical assistants (Poisson regression)

**Characteristic**		**Anxiety**^b^		**Depression**^c^	
		**PR**	**95% CI**	**PR**	**95% CI**
Any WPV^a^	Model I^d^	**1.95**	1.24-3.08	**1.84**	1.19-2.85
	Model II^e^	**1.85**	1.16-2.95	**1.72**	1.11-2.67

Prevalence ratio (PR) and 95% confidence interval (CI); number in bold = significant *p*-value < 0.05;

^a^any WPV: 12-month prevalence of verbal violence, physical violence or sexual harassment merged into a single variable (any affirmative response vs. none)

^b^generalized anxiety disorder questionnaire (GAD-2)

^c^patient health questionnaire (PHQ-2)

^d^unadjusted

^e^additionally adjusted for age and average working hours per week

### Sensitivity analysis

The sensitivity analysis exploring an association between the “frequency of verbal violence” and mental health variables suggests that the prevalence of poor mental health may increase with the frequency of the independent variable “verbal violence”. This trend was observed for anxiety (*p* = 0.055) and depression (*p* = 0.01) alike (see Additional file 2, Table S1).

## Discussion

We found that MAs in Germany regularly face different types of WPV, in particular verbal violence. This study suggests that patients are the main types of perpetrators, followed by patients’ relatives, colleagues and supervisors. Overall, only few sociodemographic and occupational correlates were associated with WPV among MAs (i.e., younger age, being single and working in a medical care center). WPV showed fairly pronounced associations with poor mental health.

### Comparison to prior research

#### Prevalences

To the best of our knowledge, these are the first estimates of the prevalences of WPV against MAs. In our study, younger MAs were most likely to report WPV. Our study sample comprised a higher proportion of older MAs compared to the overall MA population [21]. Consequently, the prevalences of WPV may even be higher than estimated in our study in the general MA population.

A prior cross-sectional study among physicians in outpatient care in Germany, examined WPV in the same setting as well as for the same reference period [10]. In that study, the 12-month prevalence of verbal violence in the practice among physicians was slightly lower (48% for verbal insults and/or abuse, and 17% for threats and/or intimidation vs 59% verbal violence in our study). The prevalences for physical violence were similar as in our study [3% mild physical violence (e.g., pushing, hassling) and 2% pronounced physical violence (e.g., hitting, kicking) vs 6% physical violence in our study]. Reported sexual harassment in the practice was higher both for female (15%) and male (5%) physicians compared to the prevalence in our study (4%) [10]. In a qualitative study, MAs reported that since the onset of the SARS-CoV-2 pandemic verbal violence was frequently experienced on the phone or patients behaved aggressively if their demands were not met immediately [34]. As many of the conflict-laden patient-related tasks are performed by MAs and as MAs are perceived to have a lower social standing than physicians, it seems plausible that MAs are more likely to experience verbal violence by patients and patient relatives compared to physicians in outpatient care settings. A European multinational study among nurses mainly working in hospital settings measured the 12-month prevalence of verbal and physical violence using a questionnaire similar to the one applied in this study [35]. The study reported a 12-month prevalence of verbal violence of 54% and 20% for physical violence. The latter prevalence was much higher than in our study. Physical violence has been found to be higher against health care workers working in hospital settings compared to outpatient settings amongst others because more time is spent with the patients and the thresholds for aggression may be lower (e.g., in psychiatric institutions) [1, 36]. Overall, comparison with the existing literature is limited as previous studies analyzed different occupational populations (mainly nurses and physicians), mainly focused on the hospital setting and assessed WPV differently.

MAs in our study identified patients as the main perpetrators, followed by patient relatives, and further colleagues and supervisors. This pattern of perpetrators has been reported by a number of studies among nurses [5, 35]. WPV perpetrated by colleagues and supervisors is generally assessed less frequently [5, 37]. However, aggression perpetrated by supervisors and colleagues was correlated with higher psychological distress among employees compared to aggressions perpetrated by outsiders [38]. This highlights that supervisors and colleagues also need to be considered in empirical studies.

#### Sociodemographic and occupational correlates of WPV

We found that younger age, was associated with experiencing WPV. In line with our observation, a meta-analysis covering health care workers working in hospitals and outpatient settings found younger age to be predictive of WPV [1]. This finding highlights that particularly younger MAs should be protected and targeted by potential prevention measures. One might assume that the younger age implies that MAs have fewer years of work experience and therefore less experience in dealing with aggressive situations [39]. However, in our study, years of work experience were not associated with any WPV.

In our study, being single was associated with more frequent reports of any WPV in the preceding 12 months. The available evidence suggests that being single is associated with a higher prevalence of experiencing physical violence [1]. A potential explanation may be that single MAs may have less social support through family, which has been found to be associated with a greater risk of experiencing violence [40].

We found the prevalence of WPV to be higher among MAs working in medical care centers compared to GP practices. One might assume that the high staff turnover rates in medical care centers may lead to poor work relationships among colleagues and with the supervisors, which was associated with WPV in a cross-sectional study among healthcare workers in Portugal [41, 42]. However, potential explanations for this association need to be further investigated.

#### WPV and mental health

No study has examined potential mental health consequences of WPV for the MA occupation. A cohort study among health care workers in Italy found non-physical violence to be predictive of poor mental health [43]. A longitudinal study among the general working population in Denmark showed that exposure to work-related violence increased the risk of depressive disorders [44]. Our observations are in line with these findings for another health care profession, though the limitation of the cross-sectional nature of our study needs to be kept in mind (see limitation section). In a sensitivity analysis, we found evidence of a potential association between the frequency of verbal violence and adverse mental health (see Additional file 2, Table S1). A prospective study among public employees from Denmark provided evidence of a dose–response relationship between frequency of workplace violence and depression [45]. Our findings suggest that such dose–response relationships, which are generally understood as one of various indicators of causality [46], may also be present among MAs.

### Limitations

The limitations of our study include, first, the cross-sectional design which does not allow determination of the potential direction of the examined associations. Second, the response rate cannot be calculated due to widespread recruitment effort and an unknown denominator. We cannot rule out that particularly motivated MAs or MAs affected by WPV have participated in this study. However, the MAs who participated in this study are fairly representative of the general MA population in Germany in terms of gender and employment status [21]. Overall, the age distribution seems also comparable, although the proportion in the age group below 40 years in our study is lower than in the overall MA population (26% vs 36%, respectively). This deviation may be of particular relevance in terms of the estimation of the prevalences, in particular of sexual harassment at work, which has been found to be more frequent in younger workers [47]. Moreover, a more adequately powered and more representative sample may have yielded more precise and more valid estimates on the association between age and WPV. Fourth, we inquired after WPV as experienced in the preceding 12 months, which may be prone to recall bias. Fifth, verbal violence and sexual harassment may overlap to some extent [3] and thus their separate assessment may be subject to reporting bias. To better clarify differences between these two types of violence, participants were given definitions (see Additional file 1). To minimize reporting bias in the association analysis, we used the combined variable “any WPV”. Sixth, correlates of WPV were analyzed with the combined WPV variable and for all perpetrators combined due to low number of cases in the subgroups and therefore limited statistical power. Correlates of the different forms of WPV and the different perpetrators could therefore not be analyzed. Finally, our study was carried out during the SARS-CoV-2 pandemic and specifically at a time when the general population in Germany was eligible for the vaccination and when the workload in outpatient care settings and the distress among practice teams was exceptionally high. This may have led to a higher than usual prevalence of verbal violence, as MAs reported that patients became more demanding and behaved aggressively, particularly on the phone (e.g., to schedule vaccination appointments, obtain test results), during longer waiting times, and due to new practice organization approaches due to SARS-CoV-2 regulations [34].

### Recommendations for future research

This study shows that WPV against MAs is a major problem in Germany. It has been suggested that WPV against healthcare workers in Germany is increasing [36]. Therefore, prospective studies are needed to monitor the prevalences and trends of WPV against MAs. Further, prospective studies would allow to clarify the direction of associations, in particular between WPV and poor health among MAs. Qualitative studies would help to gain a profound understanding of how WPV emerges in outpatient care in Germany and further how MAs perceive and handle WPV. These data could provide starting points for preventive measures that would benefit MAs and likely the entire practice team, including physicians. Moreover, the underlying structures of the higher prevalence of WPV in medical care centers compared to GP practices should be examined in future studies. In addition, future studies should be adequately powered in order to derive more precise estimates of potential WPV type-specific or perpetrator-specific correlates.

### Recommendations for practice and prevention

As prevention of WPV is important, we would like to make potential recommendations. However, our recommendations should be considered with caution due to the cross-sectional design of our study. Future longitudinal studies need to clarify the direction of the hypothesized relationships to thereby corroborate the validity of our recommendations and to inform the development and implementation of promising interventions. A potential starting point for action could be to introduce de-escalation and nonviolent communication strategies (e.g., recognition of aggressive cues and behaviors, enhancing communication skills) into the curriculum in vocational training of MAs [48]. Even though data do not show unambiguously that training opportunities reduce the incidence of WPV, such trainings may likely bring about an increased feeling of confidence, knowledge and skills among participants [48]. The implementation of such training opportunities in vocational training would thereby benefit especially the younger MAs, who have been identified as an at-risk group. Additionally, MAs could be taught in vocational school that in cases where physical violence resulted in injuries, documentation by trained physicians is recommended and necessary to protect legal interests. Moreover, specific trainings against WPV in medical care centers are needed to bring solution to workplaces, which are associated with more frequent exposure to WPV. However, it is important to mention that an open attitude towards this topic and a zero tolerance policy towards violence by the entire work team is a key prerequisite for the effectiveness of any preventive measure [36]. Moreover, formal policies and action plans for dealing with violence in the workplace might be helpful in order to provide clarity on the scope of potential actions (e.g., giving MAs the permission to expel patients or patient relatives from the practice) [49].

In the case of experienced WPV regular exchange groups with colleagues and contact points could strengthen social support networks, which may especially benefit single MAs with low social support structures. Potential health sequels such as anxiety and depressive symptoms emphasize the need for improvement of the support of MAs in case of WPV. Especially because a review described that very few health care workers who have experienced WPV actually seek professional psychological help [6]. The “Employers' Liability Insurance Association for Medical Services and Welfare Work”, of which all MAs in Germany are compulsory members, considers WPV in any form as an occupational accident if it caused physical or psychological injuries, and therefore covers the cost of care (e.g., psychological) if needed [50]. The prerequisite for this is that the incidents are reported to the employers' liability insurance association [50]. Numbers on the use of this reporting opportunity among MAs are not known. Especially in outpatient care settings, MAs may benefit when the existing support structures are promoted and their utilization is encouraged.

Finally, a WPV reporting system should be implemented in health care institutions in Germany to strengthen the reporting of WPV, as reporting of WPV among healthcare workers has been found to be low (16%) [51]. The system could in addition function as an information platform providing information on psychological counseling and possible legal action, and to develop practical solution strategies.

## Conclusion

This study is the first to report prevalences of different types of WPV against MAs in Germany. Our findings show the scope of WPV against MAs, the broad spectrum of perpetrators and correlates of WPV (e.g., younger age, being single and working in a medical care center). In addition, WPV was found to be associated with poor mental health. Further prospective studies are needed to confirm the potential risk groups for WPV or health sequels of WPV observed in our study and to devise preventive measures.

## Supplementary Information

**Additional file 1.** Questionnaire.

**Additional file 2:** **Table S1.** Association of frequencyof verbal violence experienced in the preceding 12 months with poor mental healthamong medical assistants (Poisson regression).

## Data Availability

The dataset generated and analyzed during the study is not publicly available due to privacy concern, but are available from the corresponding author on reasonable request.

## Abbreviations

CI: Confidence interval
GAD: Generalized anxiety disorder
GP: General practitioner
ILO: International Labor Organization
MA: Medical assistant
PHQ: Patient health questionnaire
PR: Prevalence ratio
VMF: Association of Medical Professions
WPV: Workplace violence

## References

[1] Liu J, Gan Y, Jiang H, Li L, Dwyer R, Lu K (2019). Prevalence of workplace violence against healthcare workers: A systematic review and meta-analysis. Occup Environ Med..

[2] International Labour Organization, International Council of Nurses, World Health Organization, Public Services International. Framework guidelines for addressing workplace violence in the health sector/ Joint Programme on Workplace Violence in the Health Sector. Geneva; 2002.

[3] Di Martino V (2003). Relationship between work stress and workplace violence in the health sector.

[4] Pompeii L, Benavides E, Pop O, Rojas Y, Emery R, Delclos G (2020). Workplace violence in outpatient physician clinics: a systematic review. Int J Environ Res Public Health.

[5] Spector PE, Zhou ZE, Che XX (2014). Nurse exposure to physical and nonphysical violence, bullying, and sexual harassment: a quantitative review. Int J Nurs Stud..

[6] Lanctôt N, Guay S (2014). The aftermath of workplace violence among healthcare workers: a systematic literature review of the consequences. Aggress Violent Behav.

[7] Gillespie GL, Gates DM, Miller M, Howard PK (2010). Workplace violence in healthcare settings: risk factors and protective strategies. Rehabil Nurs.

[8] Al-Turki N, Afify AAM, Alateeq M (2016). Violence against health workers in family medicine centers. J Multidiscip Healthc.

[9] El-Gilany AH, El-Wehady A, Amr M (2010). Violence against primary health care workers in Al-Hassa Saudi Arabia. J Interpers Violence.

[10] Vorderwülbecke F, Feistle M, Mehring M, Schneider A, Linde K (2015). Aggression and violence against primary care physicians—a nationwide questionnaire survey. Dtsch Arztebl Int.

[11] Fisekovic MB, Trajkovic GZ, Bjegovic-Mikanovic VM, Terzic-Supic ZJ (2015). Does workplace violence exist in primary health care? Evidence from Serbia. Eur J Public Health.

[12] Rincón-Del Toro T, Villanueva-Guerra A, Rodríguez-Barrientos R, Polentinos-Castro E, Torijano-Castillo MJ, de Castro-Monteiro E (2016). Aggressions towards primary health care workers in Madrid, Spain, 2011–2012. Rev Esp Salud Publica.

[13] Forrest LE, Herath PM, McRae IS, Parker RM (2011). A national survey of general practitioners’ experiences of patient-initiated aggression in Australia. Med J Aust.

[14] Magin P, Joyce T, Adams J, Goode S, Cotter G (2009). Receptionists’ experiences of occupational violence in general practice: a qualitative study. Br J Gen Pract.

[15] Grobe TG, Szecsenyi J. [Barmer physician report 2021] Barmer Arztreport 2021. 2021;218.

[16] Federal Employment Agency. [Statistics of the Federal Employment Agency: Employees subject to social insurance contributions in the occupational subgroup - medical assistants] Statistik der Bundesagentur für Arbeit: Sozialversicherungspflichtige Beschäftigte in der Berufsuntergrupp. 2019.

[17] National Association of Statutory Health Insurance Physicians, National Association of Statutory Health Insurance Funds. [Agreement on the delegation of medical services to non-physician staff in outpatient contract medical care pursuant to § 28 para. 1 sentence 3 SGB V] Vereinbarung über die Delegation ärztlicher Leistungen an nichtärztliches Personal in der ambulanten ver. 2015;11.

[18] Ruiz-Hernández JA, López-García C, Llor-Esteban B, Galián-Muñoz I, Benavente-Reche AP (2016). Evaluation of the users violence in primary health care: adaptation of an instrument. Int J Clin Heal Psychol.

[19] Cocker F, Martin A, Scott J, Venn A, Sanderson K (2012). Psychological distress and related work attendance among small-to-medium enterprise owner/managers: literature review and research agenda. Int J Ment Health Promot.

[20] Vu-Eickmann P, Loerbroks A (2017). Psychosocial working conditions of physician assistants: results from a qualitative study on occupational stress, resources, possible approaches to prevention and intervention needs. Z Evid Fortbild Qual Gesundhwes.

[21] Federal Statistical Office (Destatis). [GENESIS database: health workforce: Germany, years, employment, age groups, health professions] Datenbank GENESIS: Gesundheitspersonal: Deutschland, Jahre, Beschäftigungsverhältnis, Altersgruppen, Berufe im Gesundheitswesen. Wiesbaden, Germany; 2019.

[22] De Jager L, Deneyer M, Buyl R, Roelandt S, Pacqueu R, Devroey Di. Cross-sectional study on patient-physician aggression in Belgium: Physician characteristics and aggression types. BMJ Open. 2019;9(12):e025942. 10.1136/bmjopen-2018-025942 PMC6937035 31857295

[23] Vu-Eickmann P, Li J, Müller A, Angerer P, Loerbroks A (2018). Associations of psychosocial working conditions with health outcomes, quality of care and intentions to leave the profession: Results from a cross-sectional study among physician assistants in Germany. Int Arch Occup Environ Health.

[24] Scharf J, Vu-Eickmann P, Li J, Müller A, Wilm S, Angerer P (2019). Desired improvements of working conditions among medical assistants in Germany: a cross-sectional study. J Occup Med Toxicol.

[25] Mambrey V, Angerer P, Loerbroks A (2022). Psychosocial working conditions as determinants of concerns to have made important medical errors and possible intermediate factors of this association among medical assistants – a cohort study. BMC Health Serv Res.

[26] International Labour Organization, International Council of Nurses, World Health Organization, Public Services International. Workplace violence in the health sector country case studies research instruments. 2003. p. 1–14. https://cdn.who.int/media/docs/default-source/documents/violence-against-health-workers/wvquestionnaire.pdf?sfvrsn=9f6810a5_2&download=true. Accessed 06 Apr 2023.

[27] Eggert S, Schnapp P, Sulmann D. [Aggression and violence in informal care] Aggression und Gewalt in der informellen Pflege. Berlin; 2018.

[28] Federal Anti-Discrimination Agency Germany. ["What to do about sexual harassment in the workplace?" Guideline for employees, employers and works boards] “Was tun bei sexueller Belästigung am Arbeitsplatz?” Leitfaden für Beschäftigte. Berlin: Arbeitgeber und Betriebsräte; 2021.

[29] Schenk L, Bau A-M, Borde T, Butler J, Lampert T, Neuhauser H, et al. [A basic set of indicators for mapping migrant status. Recommendations for epidemiological practice]. Bundesgesundheitsblatt- Gesundheitsforschung- Gesundheitsschutz. 2006;49(9):853–60. 10.1007/s00103-006-0018-4 16927038

[30] Kroenke K, Spitzer RL, Williams JBW, Loewe B (2010). The patient health questionnaire somatic, anxiety, and depressive symptom scales: a systematic review. Gen Hosp Psychiatry.

[31] Plummer F, Manea L, Trepel D, McMillan D (2016). Screening for anxiety disorders with the GAD-7 and GAD-2: a systematic review and diagnostic metaanalysis. Gen Hosp Psychiatry.

[32] Löwe B, Kroenke K, Gräfe K (2005). Detecting and monitoring depression with a two-item questionnaire (PHQ-2). J Psychosom Res.

[33] Knol MJ, Le Cessie S, Algra A, Vandenbroucke JP, Groenwold RHH (2012). Overestimation of risk ratios by odds ratios in trials and cohort studies: Alternatives to logistic regression. CMAJ..

[34] Dreher A, Mambrey V, Loerbroks A (2022). Changes of working conditions and job-related challenges due to the SARS-CoV-2 pandemic for medical assistants in general practices in Germany: a qualitative study. BMC Prim Care.

[35] Babiarczyk B, Turbiarz A, Tomagová M, Zeleníková R, Önler E, Cantus DS (2020). Reporting of workplace violence towards nurses in 5 european countries – a cross-sectional study. Int J Occup Med Environ Health.

[36] Schablon A, Wendeler D, Kozak A, Nienhaus A, Steinke S (2018). Prevalence and Consequences of Aggression and Violence towards Nursing and Care Staff in Germany—A Survey. Int J Environ Res Public Health.

[37] Zhang Y, Cai J, Yin R, Qin S, Wang H, Shi X (2022). Prevalence of lateral violence in nurse workplace: a systematic review and meta-analysis. BMJ Open.

[38] Hershcovis MS, Barling J (2010). Towards a multi-foci approach to workplace aggression: A meta-analytic review of outcomes from different perpetrators. J Organ Behav.

[39] Al-Azzam M, Al-Sagarat AY, Tawalbeh L, Poedel RJ (2017). Mental health nurses’ perspective of workplace violence in Jordanian mental health hospitals. Perspect Psychiatr Care.

[40] Montesó-Curto P, Aguilar C, Lejeune M, Casadó-Marin L, Casanova Garrigós G, Ferré-Grau C (2017). Violence and depression in a community sample. J Clin Nurs.

[41] Central Research Institute of Ambulatory Health Care in Germany. [Zi-MVZ-Panel - Annual Report 2020; Economic situation, general conditions and supply in medical care centers in 2017.] Zi-MVZ-Panel - Jahresbericht 2020; Wirtschaftliche Situation, Rahmenbedingungen und Versorgung in Medizinischen Versorgungszentren im J. Berlin, Germany; 2021.

[42] Barros C, Meneses RF, Sani A, Baylina P (2022). Workplace Violence in Healthcare Settings: Work-Related Predictors of Violence Behaviours. Psych.

[43] Magnavita N (2014). Workplace violence and occupational stress in healthcare workers: A chicken-and-egg situation-results of a 6-year follow-up study. J Nurs Scholarsh.

[44] Madsen IEH, Svane-Petersen AC, Holm A, Burr H, Framke E, Melchior M (2021). Work-related violence and depressive disorder among 955,573 employees followed for 6.99 million person-years. The Danish Work Life Course Cohort study: Work-related violence and depression. J Affect Disord..

[45] Rudkjoebing LA, Hansen ÅM, Rugulies R, Kolstad H, Bonde JP (2021). Exposure to workplace violence and threats and risk of depression: a prospective study. Scand J Work Environ Health.

[46] Gordis L (2008). Epidemiology, fourth edition.

[47] Jonsdottir SD, Hauksdottir A, Aspelund T, Jakobsdottir J, Runarsdottir H, Gudmundsdottir B (2022). Risk factors for workplace sexual harassment and violence among a national cohort of women in Iceland: a cross-sectional study. Lancet Public Heal.

[48] Heckemann B, Zeller A, Hahn S, Dassen T, Schols JMGA, Halfens RJG (2015). The effect of aggression management training programmes for nursing staff and students working in an acute hospital setting. A narrative review of current literature. Nurse Educ Today..

[49] Bruce MD, Nowlin WA (2011). Workplace violence: awareness, prevention, and response. Public Pers Manage.

[50] Heidrich C, Jarisch H, Kix H-J, Küfner S, Pude W, Richter D, et al. [Prevention of violence and aggression against employees] Prävention von Gewalt und Aggression gegen Beschäftigte. Hamburg; 2018.

[51] Ferri P, Silvestri M, Artoni C, Di Lorenzo R (2016). Workplace violence in different settings and among various health professionals in an Italian general hospital: A cross-sectional study. Psychol Res Behav Manag.

