# Supplementary material for: Prevalence and correlates of workplace violence against medical assistants in Germany: a cross-sectional study
Source: BMC Health Serv Res. 2023 Apr 10;23:350. doi: 10.1186/s12913-023-09331-9 (PMC10088275; doi:10.1186/s12913-023-09331-9)
Supplement: Supplementary file 1 — Additional file 1. Questionnaire. [file 12913_2023_9331_MOESM1_ESM.docx]

**Questionnaire**

Physical violence

| What is meant by physical violence? This means, for example, have you been bitten, spat on, pushed, shoved or kicked, hit with your hand or an object? Have you been roughly handled or had someone pull on you? | |
| --- | --- |
| In the last 12 months, have you been physically attacked in your workplace? | - Yes - No |
| How were you physically attacked? (Multiple answers possible) | - Physical violence without a weapon - Physical violence with a weapon |
| Who attacked you?  (Multiple answers possible) | - Patients - Relatives of patients - Colleagues - Supervisors - Others:__________________ |
| How often have you been physically attacked in the last 12 months? | - (Almost) daily - Weekly - Monthly - Once in a quarter - Once in a year |

Verbal violence

| What is meant by verbal / psychological violence? That is, were you, for example, intimidated or threatened with words, insulted or were derogatory remarks made about you, were you yelled at or bossed around? | |
| --- | --- |
| In the last 12 months, have you been verbally abused in your workplace? | - Yes - No |
| Who verbally abused you?  (Multiple answers possible) | - Patients - Relatives of patients - Colleagues - Supervisors - Others:__________________ |
| How often have you been verbally abused in the last 12 months? | - (Almost) daily - Weekly - Monthly - Once in a quarter - Once in a year |

Sexual harassment/violence

| What is meant by sexual harassment? That is, for example, were lewd remarks made, were you touched unpleasantly on parts of your body, did the person perform sexual acts on himself or herself in your presence? | |
| --- | --- |
| In the last 12 months, do you feel you have been sexually harassed at your workplace? | - Yes - No |
| Who sexually harassed you?  (Multiple answers possible) | - Patients - Relatives of patients - Colleagues - Supervisors - Others:__________________ |
| How were you harassed? (Multiple answers possible) | - Sexually suggestive remarks and jokes - Solicitation of unwanted intimate or sexual acts (e.g., "Sit on my lap") - Intrusive or intimidating stares or suggestive glances - Unwanted touching (patting, stroking, hugging), even if the touching appears to be incidental - Sexualized assault (grabbing breasts and/or genitals) - Rape |
| How often have you been sexually harassed in the last 12 months? | - (Almost) daily - Weekly - Monthly - Once in a quarter - Once in a year |
