# Supplementary material for: Prevalence and correlates of workplace violence against medical assistants in Germany: a cross-sectional study
Source: BMC Health Serv Res. 2023 Apr 10;23:350. doi: 10.1186/s12913-023-09331-9 (PMC10088275; doi:10.1186/s12913-023-09331-9)
Supplement: Supplementary file 2 — Additional file 2: Table S1. Association of frequencyof verbal violence experienced in the preceding 12 months with poor mental healthamong medical assistants (Poisson regression). [file 12913_2023_9331_MOESM2_ESM.docx]

Table S1. Association of frequency of verbal violence experienced in the preceding 12 months with poor mental health among medical assistants (Poisson regression).

| **Frequency of verbal violence** | **Anxiety^a^** | |  | **Depression^b^** | |  |
| --- | --- | --- | --- | --- | --- | --- |
|  | **PR** | **95% CI** |  | **PR** | **95% CI** |  |
| Once in a year | 1.00 | ref |  | 1.0 | ref |  |
| Once in a quarter | 1.23 | 0.50, 3.00 |  | 1.12 | 0.46, 2.74 |  |
| Monthly | 1.06 | 0.41, 2.73 |  | 1.36 | 0.55, 3.32 |  |
| Weekly | 1.63 | 0.66, 4.05 |  | 1.30 | 0.52, 3.24 |  |
| (Almost) daily | 2.19 | 0.82, 5.82 |  | **2.81** | **1.15, 6.86** |  |

Prevalence ratio (PR) and 95% confidence interval (CI); model additionally adjusted for age and average working hours per week; number in bold = significant p-value <0.05

^a^ generalized anxiety disorder questionnaire (GAD-2);

**^b^** patient health questionnaire (PHQ-2).
